# Simian Immunodeficiency Virus-Derived Extracellular Vesicles Induce a Chronic Inflammatory Phenotype in Healthy Astrocytes Unresolved by Anti-Retroviral Therapy

**DOI:** 10.3390/pharmaceutics17111374

**Published:** 2025-10-24

**Authors:** Alison R. Van Zandt, Miranda D. Horn, Ryan P. McNamara, Tiffany A. Peterson, Nicholas J. Maness, Blake Schoest, Elise M. Frost, Yijun Zhou, Matilda J. Moström, Dirk P. Dittmer, Andrew G. MacLean

**Affiliations:** 1 Tulane National Primate Research Center, Tulane University, Covington, LA 70433, USA; avanzandt@tulane.edu (A.R.V.Z.); mdhorn@owu.edu (M.D.H.);; 2 Biomedical Sciences Training Program, School of Medicine, Tulane University, New Orleans, LA 70112, USA; 3 Tulane Brain Institute, New Orleans, LA 70118, USA; 4 Department of Microbiology and Immunology, The University of North Carolina at Chapel Hill, Chapel Hill, NC 27599, USA; 5 Lineberger Comprehensive Cancer Center, The University of North Carolina at Chapel Hill, Chapel Hill, NC 27599, USA; 6 Department of Pathology and Laboratory Medicine, School of Medicine, Tulane University, New Orleans, LA 70112, USA; 7 Louisiana Cancer Research Center, New Orleans, LA 70112, USA

**Keywords:** extracellular vesicle, exosome, astrocyte, glia, neuroinflammation

## Abstract

**Background/Objectives:** Extracellular vesicles (EVs) are key mediators of intercellular communication and are implicated in the neuropathogenesis of HIV-associated brain injury (HABI). However, their direct effects on glial cells, particularly in the context of antiretroviral therapy (ART), remain incompletely understood. **Methods:** In this study, we investigated how EVs from naïve, Simian Immunodeficiency Virus (SIV)-infected, and SIV-infected ART-treated rhesus macaques impact primary mixed glial cultures. **Results:** Through multiple, sequential applications mimicking chronic exposure, we found that EVs from SIV-infected animals significantly reduced glial expansion and induced a simplified, reactive astrocyte morphology indicative of neuroinflammatory stress. In contrast, EVs from naïve animals supported glial health. EVs derived from ART-treated animals provided partial protection from SIV-induced effects, yet still suppressed glial proliferation and failed to fully restore normal morphology. Furthermore, cytokine profiling revealed that both SIV and SIV + ART EVs induced a sustained proinflammatory secretory phenotype, characterized by elevated IL-6, IL-8, and IFN-γ. **Conclusions:** Our findings demonstrate that systemically circulating EVs in SIV infection are potential drivers of glial dysfunction. The persistence of these pathogenic EV effects despite ART suggests a vesicle-mediated mechanism that may contribute to chronic neuroinflammation and cognitive impairment in virally suppressed individuals.

## 1. Introduction

Extracellular vesicles (EVs) are lipid-bound nanoparticles that mediate intercellular communication by transporting proteins, lipids, and nucleic acids. They play pivotal roles in immune regulation, cellular signaling, and the pathogenesis of neuroinflammatory diseases, including those associated with human immunodeficiency virus (HIV), simian immunodeficiency virus (SIV) and other viruses [1,2,3]. EVs contribute to viral persistence, immune modulation, and neuroinflammation, yet their specific effects on glial cells—key mediators of neuroimmune responses—remain incompletely understood [1,2,3].

Glial cells, including astrocytes, microglia, and oligodendrocytes, are highly responsive to environmental signals, including those delivered by EVs [4]. In the context of lentiviral infection, glial dysfunction and chronic activation are central drivers of HIV-associated brain injury (HABI), a neuroinflammatory condition marked by synaptic loss, neuronal damage, and cognitive decline [5,6,7]. Persistent viral infection leads to the continuous release of viral proteins and inflammatory mediators that amplify glial activation, exacerbating neuropathology [8,9]. Given the established role of EVs in immune signaling, exploring their impact on glial cells is critical for understanding the mechanisms of HABI and identifying new therapeutic targets.

Generally, EV cargo composition reflects both the physiological and pathological state of the producing cell [3,10,11,12,13]. In lentiviral infections, EVs are enriched in viral proteins such as Nef, alongside host-derived factors, microRNAs, and cytokines [2,14,15,16,17,18,19,20,21]. EVs thus act as vehicles for viral and host-derived signals, capable of modulating immune responses and altering recipient cell function [22]. Notably, SIV infection drives a shift in EV content toward a proinflammatory profile, including increased levels of inflammatory cytokines, viral RNAs, and regulatory microRNAs that further promote immune dysregulation [1,3]. In HIV and SIV infection, EV cargo does not fully normalize even under suppressive antiretroviral therapy (ART). Viral proteins, such as Nef, remain detectable in circulating EVs despite ART, suggesting an ongoing contribution to immune activation and neuroinflammation [1]. Thus, we hypothesized that EVs obtained from (a) naïve, (b) SIV-infected, and (c) SIV-infected and ART-treated animals possess distinct capacities to induce glial activation, inflammation, and neurotoxicity. Understanding these differences is crucial to clarifying EV roles in HIV pathogenesis.

Here, we investigate how EVs derived from naïve, SIV-infected, and SIV+ ART-treated macaques differentially regulate glial morphology, survival, and cytokine production in vitro. This study directly addresses the interplay between EV cargo and glial activation to advance our understanding of EV-driven neuropathology in lentiviral infections. While EVs hold therapeutic potential as drug delivery vehicles or modulators of immune responses, their role in perpetuating or mitigating neuroinflammation remains unclear. EVs from healthy cells may confer neuroprotection [23,24], whereas EVs from diseased states can propagate inflammatory cascades and neuronal injury [4,5,7,12].

## 2. Methods

### 2.1. Isolation and Characterization of EVs

Plasma from several NIH-funded studies were made available at necropsy. Although no animals were specifically used for this study, all animal studies are approved by Tulane’s Institutional Animal Care and Use Committee.

EVs were isolated from the whole blood of three groups of animals, naïve, SIV-infected, and SIV-infected + ART-treated, using the affinity-purification methods as described by McNamara et al. [25]. EV doses were equalized by particle number (NTA) so that each application delivered 1 × 10^10^ particles/mL to the replacement volume.

### 2.2. Affinity Purification of Extracellular Vesicles

Endocytic-competent extracellular vesicles (EVs) were affinity-purified from primary fluids using a previously validated method [25]. In brief, primary fluids were successively passaged through a 0.45 µm and a 0.22 µm vacuum filtration apparatus (Thermo Fisher 1690045 and 5690020, Waltham, MA, USA) or Luer-lock syringes (Fisher 149554959) coupled to 0.45 µm and a 0.22 µm filtration membranes (Fisher 13-100-197 and 25-244). Large volume fluids (>40 mL) were volumed up in 1X PBS (Gibco 10010023, Waltham, MA, USA) to 400 mL and passaged through the AKTA Flux S (General Electric 29038437, Boston, MA, USA) tangential flow filtration apparatus equipped with a 750 kDa hollow-fiber cartridge (General Electric UFP-750-E-4X2MA).

All clarified fluids were then precipitated with 40 mg/mL of polyethylene glycol 8000 (PEG, Thermo Fisher 156–500) at 4 °C overnight. The precipitates were spun down at 1200× *g* at 4 °C for 1 h and the total EV pellets were resuspended in 1X PBS. Total EVs were assayed for concentration and size by nanoparticle tracking analysis (see below, and Supplemental Figure S1). The total EV slurry was then incubated with 50 ng/mL of RNase A (Promega A7973, Madison, WI, USA) and DNase (M6101) and labeled with the membrane-intercalating dye DiI (Thermo Fisher D3911) or CellMask™ plasma membrane stain (Thermo Fisher C10046) at 37 °C for 30 min. The mixture was then passaged through a HiTrap Capto Core 700 column (General Electric 17548151). Fluorescent fractions were pooled and then added to anti-CD81 magnetic beads (Thermo Fisher 10622D) and allowed to bind at 4 °C overnight. Beads were washed with 500 µL of 1X PBS for a total of 3 washes and then CD81+ EVs were eluted from the beads with 100 µL of 200 mM Glycine pH = 2.0 at 37 °C for 15 min. The eluted material was then transferred to a separate tube containing neutralization buffer (100 mM Tris-HCl pH = 7.0 in 1X PBS). The CD81+ affinity-purified EVs were then analyzed by nanoparticle tracking analysis for concentration.

### 2.3. Nanoparticle Tracking Analysis

Total EVs and CD81+ affinity-purified EVs were analyzed by nanoparticle tracking analysis (NTA) using the ZetaView from Particle Metrix (Holly Springs, NC, USA). The instrument was calibrated with manufacturer-supplied 102 nm polystyrene beads (dilution of 1:250,000 in dd H_2_O) before each run. Upon successful calibration, the instrument, samples were diluted in ddH_2_O until concentration was in the linear range of detection. Videos were taken using three cycles in 11 positions to determine particle concentration and size distribution of each sample. Statistical groupings were achieved via analysis of variance (ANOVA) and students *t*-test using R studio v 3.5 (Boston, MA, USA) or Prism v 6 (Prism, Graph Pad, La Jolla, CA, USA) followed by pairwise *t*-test for significant differences (*p* < 0.05).

### 2.4. Mixed Glial Culture Establishment

**Figure 1:**
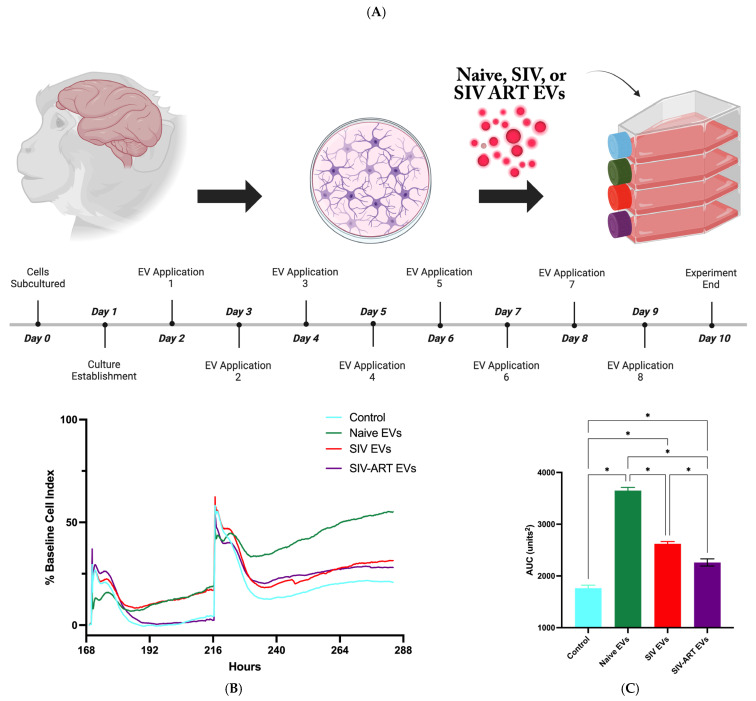
Experimental design and EV effects on glial viability and adhesion. (**A**) Primary mixed glial cultures were prepared from rhesus macaque brain tissue, plated on day 0, and stabilized for 24 h, followed by treatment with EVs isolated from naïve, SIV-infected, or SIV-infected, ART-treated animals, with eight sequential EV applications administered on days 2–9; cultures were maintained in exosome-depleted fetal bovine serum media throughout, and endpoints were assessed on day 10. (**B**) Real-time impedance monitoring demonstrated that all EV treatment groups increased impedance relative to control cultures fed exosome-depleted media, with naïve EVs producing the greatest increase over time, and two representative EV application points indicated. (**C**) Quantification of the area under the curve (AUC) revealed significant differences between groups (* *p* < 0.0001 by one-way ANOVA of all the groups followed by Bonferonni’s correction), with naïve EV-treated cultures showing the highest AUC, consistent with enhanced cellular adhesion and/or proliferation, while SIV EV and SIV + ART EV treatments produced intermediate AUC values that remained elevated compared to untreated controls; data are presented as mean ± SEM.

Primary mixed glial cultures (astrocytes +/− other glia) were established from the frontal lobe of research naïve rhesus macaques following standard protocols previously described by our laboratory [26]. As demonstrated by the timeline in Figure 1A, cells were fed twice weekly with astrocyte media containing exosome-depleted fetal bovine serum until subcultured and split into experimental groups (for xCELLigence studies) or allowed to adhere to flasks for 48 h prior to the addition of EVs (1 × 10^10^ EVs per mL) every 24 h for the duration of the study.Cultures were maintained in flasks and monitored for cell growth and process formation using phase-contrast microscopy. Flasks were grouped into the following experimental conditions:

- Group A: control group (astrocyte media containing exosome-depleted fetal bovine serum).
- Group B: EVs derived from healthy SIV-negative rhesus macaques (RMs).
- Group C: EVs derived from SIV-infected untreated RMs.
- Group D: EVs derived from SIV-infected ART-treated RMs.

### 2.5. xCELLigence Assay

Cell adhesion and proliferation were measured and plotted automatically using an xCELLigence RTCA analyzer (Agilent, Santa Clara, CA, USA) as previously described [26,27,28]. Each E-Plate was equilibrated by adding 50 μL of complete media to each well and incubating for 15 min at 37 °C in 5% CO_2_ before background impedance was established. 20,000 cells suspended in 100 μL of astrocyte media were applied to each well, with measurements taken at 5 min intervals. Experimental conditions were performed in quadruplicate as recommended in the technical manual of the RTCA instrument. A total of 24 h after plating, wells were treated with control media or media containing 1 × 10^10^ EVs per mL derived from naïve, SIV-infected, or SIV-infected and ART-treated animals. Two media replacements and EV applications were performed. Traces were plotted and analyzed using the installed software. (version 2.0)

### 2.6. Imaging and Analysis of Cell Complexity

At each media change, ten non-overlapping fields per flask were imaged with a phase-contrast microscope to assess cell counts and process development and analyzed using ImageJ software (https://imagej.net, version 1.54p). Quantification was performed blinded to treatment. Cells were included in the analysis if they met the following criteria: (1) the entire cell was within the borders of the microscopic image, and (2) the cell diameter was greater than 5 μm. Buds and processes were included if the process length was greater than 1 μm in length. To account for flask-to-flask variation, cell and branch counts from replicate flasks of the same animal were combined for further analysis. Morphological analysis of process development was performed by quantifying the proportion of cells exhibiting one, two, or three or more cellular processes. For each time point, the number of cells within each process category was divided by the total number of cells counted, yielding the percentage distribution of process complexity across the culture. All cell counts and process quantifications were performed manually using Fiji ImageJ (version 1.54p) software based on phase-contrast micrographs captured at each time point.

### 2.7. Cell Survival and Assessments of Cell Growth

Cell survival was assessed by calculating the ratio of the total cell count at each time point to the baseline cell count measured on the second day in culture (DIC). Survival data were expressed as the percentage of cells remaining relative to this baseline, providing a normalized metric of cell persistence over time.

### 2.8. Quantification of Senescence-Associated Secretory Phenotype Proteins

Supernatants were collected from cultures 24 h after each EV application, immediately prior to the subsequent application, and stored at −20 °C until analysis. Resulting solutions were analyzed for the presence of senescence-associated secretory phenotype (SASP) markers using an eleven-plex immunoassay designed for the detection of cytokines in non-human primate cells by Assay Genie (Dublin, Ireland) [29]. The eleven-plex contained: interleukin 1b (IL-1b), interleukin 10 (IL-10), Tumor Necrosis Factor-α (TNF-α), Interferon γ (IFN-γ), Interleukin 6 (IL-6), Interleukin 12 p70 (IL-12 p70), Interleukin 8 (IL-8), Interleukin 17A (IL17A), Vascular Endothelial Growth Factor (VEGF), Monocyte Chemotactic Protein 1 (MCP-1), Monocyte Chemotactic Protein (MCP-3). Fibroblast Growth Factor-B (FGF-B) was run as a separate cytokine.

All samples were examined in duplicate, and values were averaged for subsequent statistical analysis.

### 2.9. Statistical Analysis

All statistical analyses were performed using GraphPad Prism 9. Data are presented as mean ± standard error of the mean (SEM) unless otherwise indicated. Normality of data distributions was assessed using the Shapiro–Wilk test. For experiments with normally distributed data, parametric analyses were conducted; nonparametric alternatives were applied for data violating normality assumptions. For comparisons involving more than two experimental groups, a one-way analysis of variance (ANOVA) was employed, followed by post hoc tests for multiple comparisons.

xCELLigence impedance curves’ AUC values were analyzed by one-way ANOVA followed by Bonferonni’s correction; time point morphology comparisons were performed by one-way ANOVA with Tukey’s post hoc test.

Heatmap data were tested by multiple unpaired *t*-tests with Benjamini–Hochberg false discovery rate (FDR) control (q < 0.05).

To further assess group differences at specific endpoints, one-way ANOVA with Tukey’s post hoc test was also performed at the final application (day 8) for each cytokine. In addition, multiple unpaired *t*-tests with FDR correction (q < 0.05) were used to evaluate pairwise differences between treatment groups at individual time points.

Graphical representations were prepared in GraphPad Prism and matplotlib (Python version 3.13.0).

## 3. Results

### 3.1. EVs Acutely Enhance Glial Adhesion and Monolayer Impedance

All cultures for this study were derived from research naïve animals obtained at necropsy at Tulane National Primate Research Center (TNPRC). We have recently shown that the HIV protein Tat has rapid and reversible impacts on glial adhesion and activation as measured by real time impedance [30]. To investigate the impact of SIV-associated EVs on glial function, we first assessed their acute effects on the adhesion and integrity of established glial monolayers using the xCELLigence real-time cell analysis system. In these cultures, which had been grown for 24 h prior to treatment, the addition of EVs from all three donor groups (naïve, SIV, SIV + ART) led to a rapid and marked increase in the cell index (CI) compared to control cultures maintained in EV-depleted media (Figure 1B). This result demonstrates that systemically derived EVs contain bioactive factors that promote glial adhesion and support the integrity of an established monolayer.

However, the magnitude of this supportive effect was dependent on the disease state of the EV source. Quantification of the cumulative response via area under the curve (AUC) analysis (Figure 1C) revealed that naïve EVs provided the most robust support, yielding a significantly higher AUC than EVs from both SIV-infected (*p* < 0.0001) and SIV + ART-treated (*p* < 0.0001) animals. Interestingly, ART treatment did not restore this function; EVs from SIV + ART animals resulted in the lowest AUC among the treated groups. These findings suggest that while all EVs can provide acute adhesive support, the cargo of EVs from SIV-infected animals is functionally impaired in this capacity.

### 3.2. Repeated SIV EV Exposure Impairs Glial Proliferation and Survival

Having established the acute effects on mature monolayers, we next sought to determine the long-term impact of sustained EV exposure on newly established, proliferating glial cultures. Cultures from SIV-infected macaques do not proliferate [30]. Our recent study showed this may be mediated, in part, by preventing astrocytes from extending processes. Thus, primary cultures were treated repeatedly with EVs every 24 h for eight days, and cell number was monitored over time.

**Figure 2:**
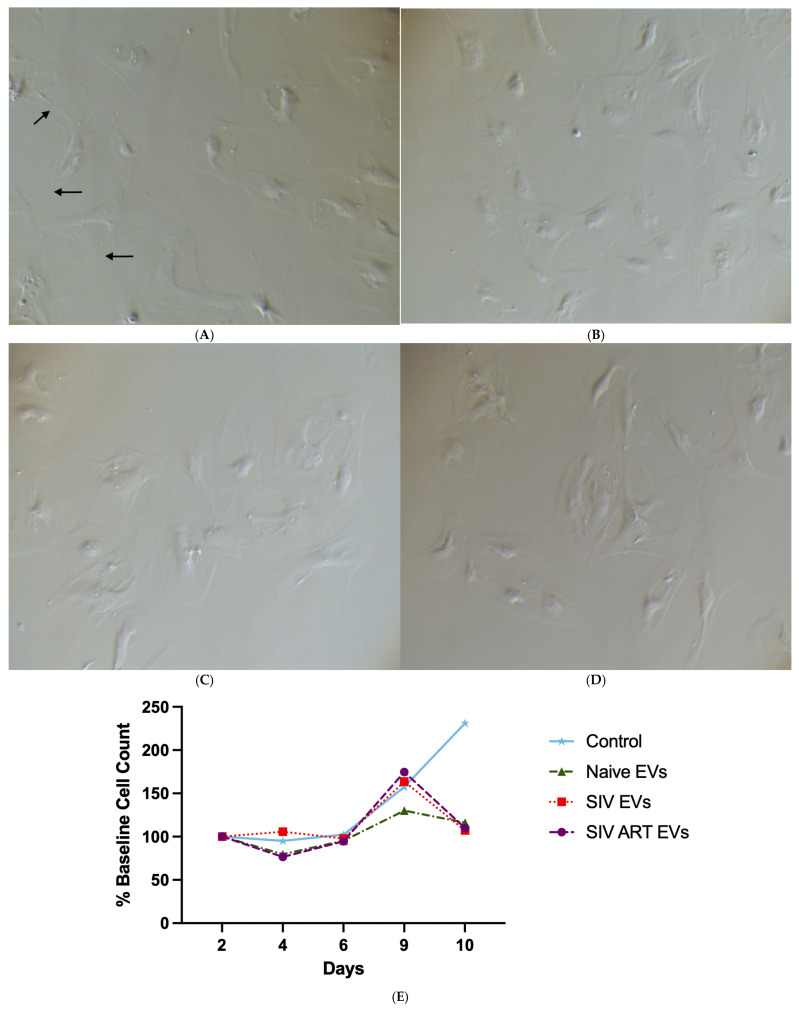
Representative phase-contrast images of primary mixed glial cultures at day 7 following EV treatment. Control glia cultured in exosome-depleted fetal bovine serum (FBS) media displayed high cell density and characteristic process formation ((**A**), arrows), consistent with the highest percentage of baseline cell count relative to day in culture (DIC). Treatment with naïve EVs showed moderate cell density and process complexity, aligning with the intermediate baseline cell count trajectory (**B**). Glia treated with SIV EVs exhibit reduced cell density and disrupted morphology (**C**), with SIV ART EV treatment demonstrate the lowest cell density and minimal process formation (**D**). Time-course graph showing percentage of baseline cell count relative to DIC 2 over the next eight days (**E**). All three EV treatments had significantly fewer cells at ten days in culture (*p* ≤ 0.0176 for naïve EVs, and *p* ≤ 0.001 for the other two groups).

Phase-contrast imaging on day seven (Figure 2A–D) revealed clear reductions in cell density with EV-treated cultures compared to controls, suggesting impaired expansion. This was confirmed by quantitative counts over the 10-day period (Figure 2E). Control cultures grown without EV exposure nearly doubled in number, whereas cultures treated with naïve EVs remained close to baseline (*p* = 0.0176), indicating a stable but non-expanding state. By contrast, chronic exposure to both SIV EVs and SIV + ART EVs not only suppressed proliferation but led to a net decline in cell numbers by day 10, demonstrating a sustained cytotoxic or growth-arresting effect not rescued by ART (*p* ≤ 0.001).

### 3.3. Repeated SIV EV Exposure Disrupts Astrocyte Morphological Complexity and Process Elaboration

**Figure 3:**
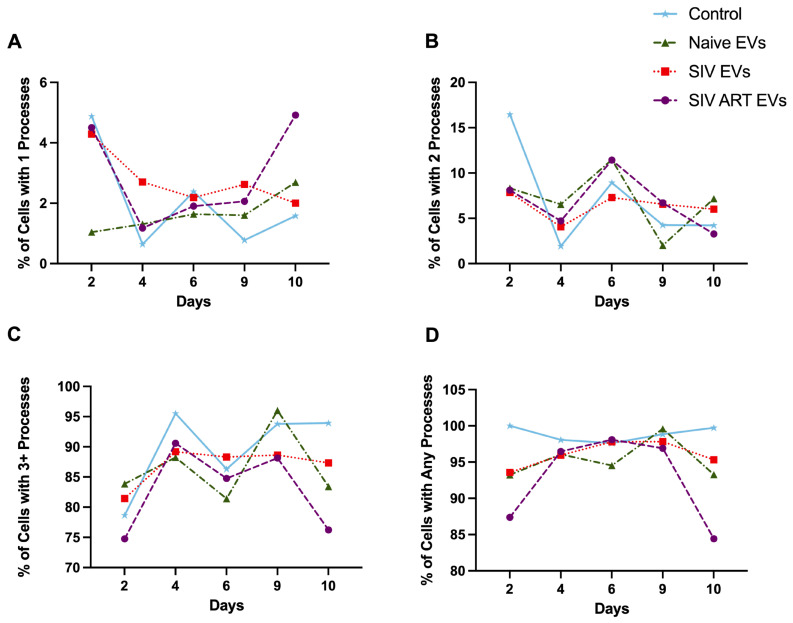
Repeated EV exposure reduces glial morphological complexity. Cultures with only one process were more initially frequent following SIV EV treatment, although SIV ART EVs induced this more simple morphology later indicating a progressive loss of complexity (**A**). Cultures exposed to SIV EVs showed a significant reduction in cells with higher-order branching, with a corresponding increase in simpler morphologies. Cells with two processes were moderately affected, with SIV EVs decreasing their proportion relative to controls, while naïve EVs maintained an intermediate distribution (**B**). Glia with ≥3 processes were most abundant in control cultures across applications 2, 4, 6, and 8 (**C**). In contrast, Cells lacking processes altogether were most prevalent in SIV EV–treated cultures, whereas ART EVs produced intermediate effects between SIV and naïve conditions (**D**). Data are presented as mean ± SEM; one-way ANOVA with Tukey post hoc at each time point, *p* < 0.05 vs. control.

To quantify these morphological changes, we analyzed the distribution of process complexity across eight EV applications (Figure 3). There were no significant changes between the treatments in the number of cells displaying 2 processes (Figure 3B). Control and naïve EV-treated astrocytes consistently maintained a complex, stellate morphology, with the majority of cells exhibiting three or more processes through nine days in culture (Figure 3C). Conversely, SIV EV exposure induced a significant and progressive simplification of astrocyte morphology, marked by a decrease in cells with ≥3 processes and a corresponding increase in cells with one or no processes (Figure 3C,D; p < 0.05). ART treatment only partially rescued this phenotype, resulting in an intermediate morphology. Collectively, these data demonstrate that SIV EVs drive astrocytes toward a reactive, simplified phenotype characterized by process retraction and a rounded, amoeboid appearance, an effect that is not fully reversed by ART.

### 3.4. Repeated SIV EV Treatment Induces Immediate Secretion of Inflammatory Cytokines: Limited Attenuation by ART

**Figure 4:**
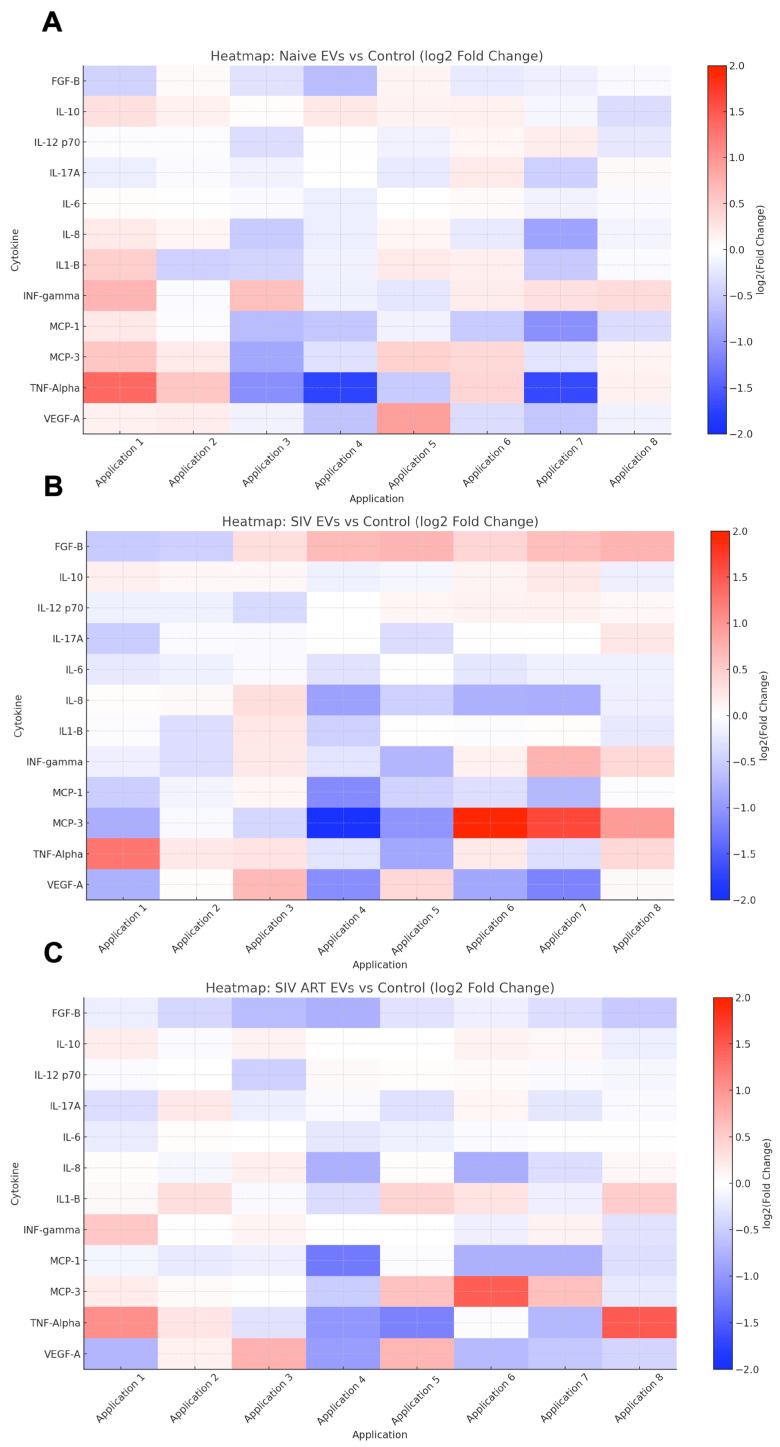

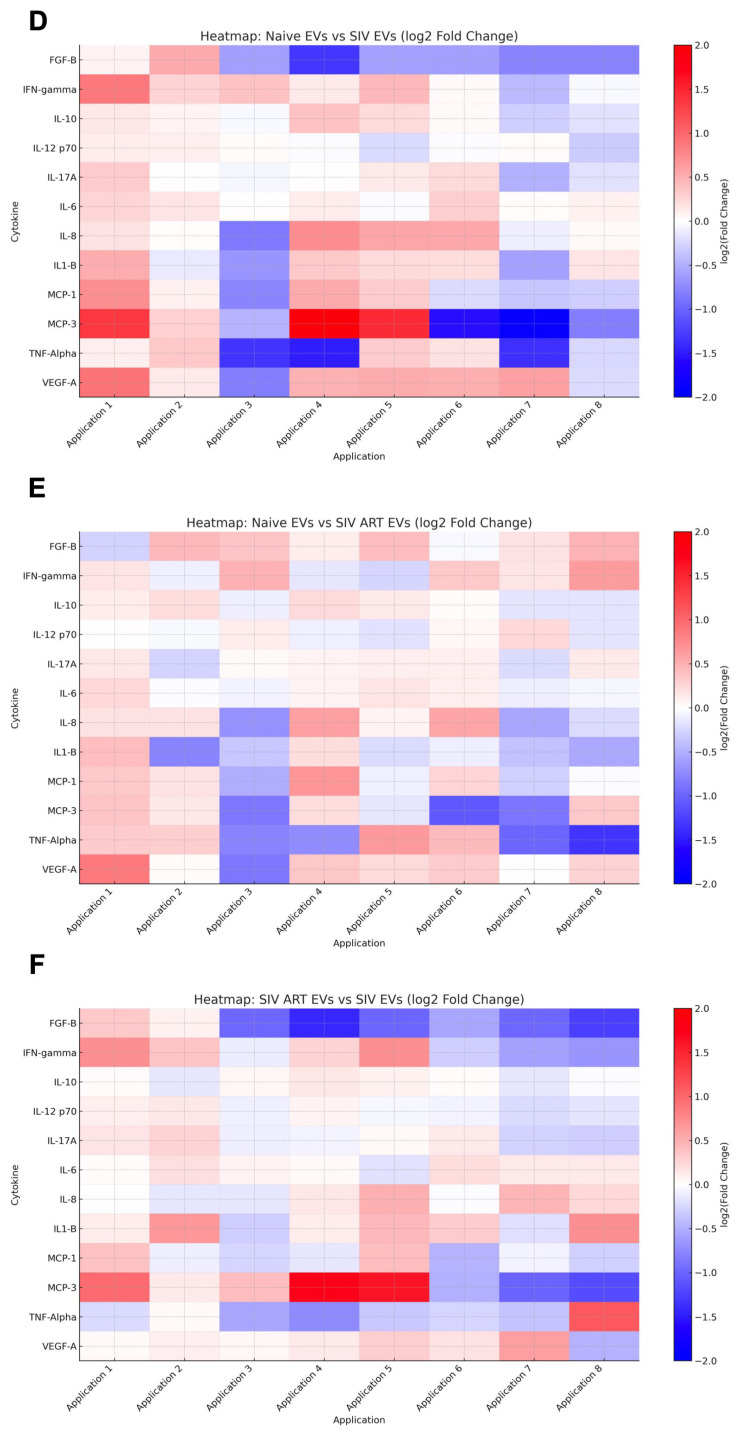
EVs from different SIV disease states differentially modulate glial cytokine secretion across repeated exposures. Heatmap visualization of cytokine secretion profiles from primary mixed glial cultures treated with EVs derived from (**A**) naïve (uninfected) animals, (**B**) SIV-infected animals, and (**C**) SIV-infected animals receiving antiretroviral therapy (ART), compared to vehicle-treated controls. Further comparisons were performed between naïve EVs and SIV EVs (**D**), naïve EVs and SIV ART EVs (**E**), and SIV ART EVs and SIV EVs (**F**).Cytokine concentrations were measured in culture supernatants prior to each successive EV application (Application 1–8) and expressed as log2 fold-change versus baseline (control). Red indicates increased cytokine levels; blue indicates reduced levels relative to control. Each column represents a cytokine measurement prior to the indicated EV application.

Heatmap analysis of secreted cytokine profiles revealed treatment-dependent modulation of astrocyte inflammatory responses following EV exposure (Figure 4). Cytokines were measured in culture supernatants immediately prior to each EV application, providing a temporal profile of secretion at baseline and post-treatment.

There were distinct patterns of proinflammatory cytokines (e.g., TNF-α, IL-6, IL-1β), anti-inflammatory cytokines (e.g., IL-10), and neurotrophic factors (FGF-B) dependent on the status of the EV donor. Notably, SIV-derived EVs (B) induced sustained proinflammatory cytokine elevation, while EVs from ART-treated SIV animals (C) exhibited partial attenuation of these responses over repeated applications. We also compared patterns between naïve EVs and SIV EVs (D), naïve EVs and SIV ART EVs (E), and SIV ART EVs and SIV EVs (F). Distinct increases in levels of MCP-3 and IL-8 were noted with SIV EVs, combined with decreased FGF-B secretion. Group differences per cytokine and time point were tested against control with multiple unpaired *t*-tests and Benjamini–Hochberg FDR control (q < 0.05).

**Figure 5:**
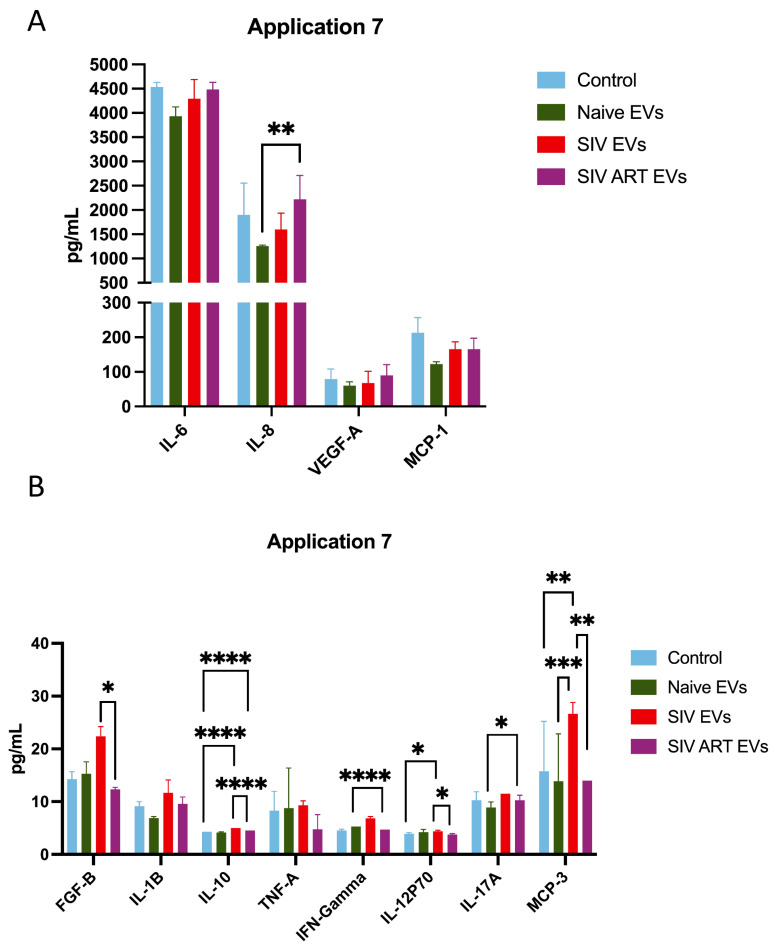
Selected cytokine trends over repeated EV exposure. Line graphs showing concentrations of key cytokines (IL-6, IL-8, VEGF-A MCP-1 (**A**), IFN-γ, IL-10, (**B**)) across 8 EV applications for control, naïve EV, SIV EV, and ART EV groups. SIV EVs induce progressive cytokine elevation; ART EVs exhibit partial suppression relative to SIV EVs but remain elevated compared to controls (mean ± SEM). Statistical significance was determined by two-way repeated-measures ANOVA with Bonferroni correction (*p* < 0.05 *, *p* < 0.01 **, *p* < 0.005 ***, *p* < 0.001 ****).

Naïve EVs induced minimal changes, maintaining a cytokine milieu similar to controls. In contrast, SIV EVs drove a sustained proinflammatory secretory phenotype, characterized by persistent upregulation of IL-1β, TNF-α, MCP-1, and IL-6. At later time points (e.g., Application 7; Figure 5), these differences became more pronounced. SIV EV treatment was associated with robust increases in IFN-γ and IL-12p70, while ART did not normalize this inflammatory profile, as SIV + ART EVs also produced marked elevations in these cytokines (*p* ≤ 0.001, and *p* = 0.0301, respectively).

Interestingly, both SIV and SIV + ART EV groups displayed a late-stage rise in IL-10, consistent with induction of a compensatory or immunoregulatory feedback loop (*p* ≤ 0.001). In addition, SIV + ART EVs uniquely induced higher levels of IL-17A compared to other groups (*p* ≤ 0.05). Finally, SIV EVs induced elevated levels of MCP-3 compared with all other treatments (*p* ≤ 0.01), in agreement with our earlier studies showing astrocyte expression of MCP-3 in SIV encephalitis [31]. Collectively, these findings indicate that SIV-associated EVs reprogram astrocytes toward a chronic, mixed inflammatory state that persists despite ART intervention.

## 4. Discussion

This study demonstrates that extracellular vesicles (EVs) derived from SIV-infected and SIV-infected-ART-treated animals exert distinct effects on primary mixed glial cultures compared to EVs derived from naïve animals, altering astrocyte adhesion, morphology, survival, and inflammatory signaling. ART only partially ameliorated the SIV effect. By systematically comparing the effects of EVs from naïve, SIV-infected, and SIV + ART animals (all with suppressed virus), we provide evidence that EV-mediated mechanisms can contribute to glial dysfunction and neuroinflammation relevant to HIV/SIV neuropathogenesis.

Our findings align with the growing recognition that EVs and their contents act as potent mediators of intercellular communication in neuroinflammation [12,32]. EVs released from virally infected cells carry viral proteins, host-derived cytokines, and microRNAs that each can modulate immune responses and cellular phenotypes in recipient cells [11,25]. In HIV/SIV infection, EVs have been implicated in blood–brain barrier dysfunction, CNS invasion, and propagation of neuroinflammatory cascades [1,33]. However, our study goes beyond associating EVs with neuroinflammation to demonstrating direct effects of highly purified EVs derived from SIV-infected macaques on multiple aspects of astrocyte biology.

We observed that SIV EVs impaired astrocyte adhesion and proliferation, evidenced by reduced impedance and a failure of glial expansion over time. Notably, despite reduced expansion over time, we observed limited overt cytotoxicity in the short term, indicating a shift toward growth suppression rather than frank cell death. One could also interpret naïve EVs as suppressing the over-proliferation seen in controls. The distinction is important: if the near-doubling in controls is non-physiological, perhaps stabilizing at baseline (as with naïve EVs) is desirable. Future studies could include viability assays including LDH release, markers of apoptosis, and/or live dead staining.

A critical finding of this study is the divergent impact of EVs in our two assay systems. While SIV-EVs were detrimental in long-term proliferating cultures, they provided acute adhesive support to established monolayers, albeit less effectively than naïve EVs. This apparent paradox likely reflects the distinct biological processes being measured. The long-term assay captures the cumulative, pathogenic effects of internalized EV cargo on astrocyte cell cycle, senescence, and survival. In contrast, the acute xCELLigence assay measures the immediate biophysical impact of EV surface ligands on cell–matrix adhesion. We interpret this to mean that while the overall SIV-EV cargo is pathogenic over time, its surface still carries molecules that can engage cellular adhesion machinery, highlighting a complex, multifaceted role for EVs in modulating glial function.

Morphometric analyses established that control astrocytes retained a high degree of process complexity, with most cells exhibiting three or more processes, many extending beyond twice the diameter of the soma. The dense overlap of these long processes formed a tightly interconnected mesh across the monolayer. Rounded or process-retracted cells were rare, indicating the absence of glial activation or reactive morphology under baseline conditions. This homeostatic morphology provides a critical baseline, distinguishing normal astrocytic features from those induced by EV-treated groups. In our control cultures, astrocytes exhibited a robust, complex morphology consistent with their prototypical homeostatic state in vitro [26,28,31,34,35,36]. Astrocytic morphology was simplified under SIV EV treatment, with fewer and shorter processes, consistent with morphologic features of reactive gliosis [11,37] and senescence-associated changes [38,39,40], aligning with prior observations [11,37].

The temporal dynamics of EV-cell interactions have emerged as a key factor in determining phenotypic changes in experimental systems. Multiple applications of KSHV-associated EVs lead to sustained changes in recipient cells, including modulation of activation markers, enhancement of apoptosis, and altered susceptibility to infection [2], affirming the notion that EV exposure is cumulative, involving both a response to immediate uptake and gradual cellular reprogramming across successive exposures. Our study builds upon this concept by applying multiple rounds of EV treatment to primary mixed glial cultures, aiming to capture both acute and sustained effects of EV signaling. We hypothesize that a single EV exposure may underestimate the chronic signaling environment present in vivo, whereas repeated exposures more accurately model the sustained impact of circulating EVs during infection and treatment.

Senescent astrocytes are characterized by flattened morphology, reduced proliferative capacity, and the acquisition of a proinflammatory secretory phenotype (SASP), marked by increased IL-6, IL-8, MCP-1, and other cytokines [29,41]. The cytokine profile we observed under SIV EV treatment parallels this senescence-associated signature, suggesting that EV exposure may drive senescence-like activation of astrocytes, contributing to chronic neuroinflammation. This phenotype is seen by other viral infections and by oxidative stress which can promote senescence via EV-mediated signaling [11,13].

The partial preservation of morphology and function in SIV + ART EV-treated cultures suggests that ART may attenuate but not fully reverse SIV-EV-induced senescence-like phenotypes. In our study, ART treatment did not reverse the SIV-EV-induced impairment in glial proliferation. This pattern diverges from prior findings with the presence of THC [30], where ART mitigated SIV- or HIV-induced impairments, suggesting potential differences in ART responsiveness depending on the experimental context or injury signal. This study aligns with findings that even under ART, HIV infection is associated with increased cellular aging and senescence in CNS cells [42,43,44]. Our results point to EVs as a potential factor of “inflammaging” and glial senescence in the HIV/SIV-infected brain [41]. This has profound clinical implications, suggesting that even in virally suppressed individuals, circulating EVs may act as persistent drivers of the chronic neuroinflammation that underlies HIV-associated neurocognitive disorders (HAND).

Taken together, these findings outline a trajectory of EV-mediated glial dysregulation, transitioning from a quiescent, morphologically complex, immunologically balanced state under naïve EV exposure to a senescent, morphologically simplified, and proinflammatory phenotype under SIV EV influence. ART partially attenuates these effects but fails to restore full homeostasis, potentially prolonging low-level neuroinflammatory deficits and supporting the hypothesis that EV-mediated signaling contributes to chronic glial dysfunction and neuroinflammation despite complete viral suppression [1,13,33].

Our findings are consistent with broader evidence implicating EVs in viral immune evasion, CNS entry, and chronic neuroinflammation [3,32]. EV-induced senescence has also been described in aging and neurodegenerative contexts, whether the same mechanisms apply in neuroHIV and other neurodegenerative diseases remains to be proven [29,41]. Irrespective of the mechanism, modulation of EV biogenesis, cargo, or uptake represent novel intervention points to mitigate chronic neuroinflammation and glial senescence in HIV/SIV neuropathology [37,43]. Future research should focus on identifying specific EV cargo that drive senescence-like activation, evaluating EV biomarkers of glial senescence, and testing interventions aimed at restoring glial homeostasis.

In summary, this study provides evidence that EVs from SIV-infected and ART-treated animals promote astrocyte morphological simplification, inflammatory activation, and features consistent with a senescence-like phenotype. These EV-driven alterations likely contribute to persistent neuroinflammation and glial dysfunction underlying HIV-associated neurocognitive disorders, offering insights into potential therapeutic targets. While our study robustly demonstrates the functional impact of disease-state-specific EVs, a limitation is that the specific viral or host cargo molecules driving these phenotypes were not identified. Future work should focus on proteomic and transcriptomic analysis of these EV populations to pinpoint the precise drivers of astrocyte dysfunction.

## 5. Conclusions

This study reveals EVs as critical mediators of glial dysfunction in SIV-associated neuroinflammation, with distinct disease-state-dependent effects on astrocyte viability, morphology, and cytokine signaling. While EVs from naïve animals preserved glial homeostasis, EVs from SIV-infected animals disrupted astrocyte survival, suppressed normal cytokine release, simplified process architecture, and induced a sustained proinflammatory phenotype. EVs from ART-treated animals provided partial protection yet retained inflammatory and angiogenic signaling, reflecting an incomplete restoration of EV function under antiretroviral therapy.

These findings provide mechanistic insight into how EV cargo evolves during infection and treatment, directly modulating glial health through inflammatory and structural pathways. Notably, the persistence of pathogenic EV signaling despite ART suggests a mechanism for chronic neuroinflammation and neurocognitive impairment in virally suppressed individuals.

From a translational perspective, this work identifies EVs as both biomarkers and potential therapeutic targets for HIV-associated neurocognitive disorders. Targeting EV biogenesis, release, or cargo—whether by modulating their production, neutralizing pathogenic components, or blocking uptake in glial cells—represents a promising avenue for mitigating CNS inflammation and preserving neurological function in HIV/SIV infection. Future studies should prioritize the molecular dissection of EV content and the development of interventions aimed at disrupting these vesicle-mediated pathogenic signals.

## Data Availability

The original contributions presented in this study are included in the article. Further inquiries can be directed to the corresponding author.

## Supplementary Materials

The following supporting information can be downloaded at: https://www.mdpi.com/article/10.3390/pharmaceutics17111374/s1, Figure S1: Purification and characterization of extracellular vesicles.
