# Supplementary material for: Simian Immunodeficiency Virus-Derived Extracellular Vesicles Induce a Chronic Inflammatory Phenotype in Healthy Astrocytes Unresolved by Anti-Retroviral Therapy
Source: Pharmaceutics. 2025 Oct 24;17(11):1374. doi: 10.3390/pharmaceutics17111374 (PMC12655024; doi:10.3390/pharmaceutics17111374)
Supplement: Supplementary file 1 [file pharmaceutics-17-01374-s001.zip › pharmaceutics-3895808-Supplemental Figure 1.pdf]

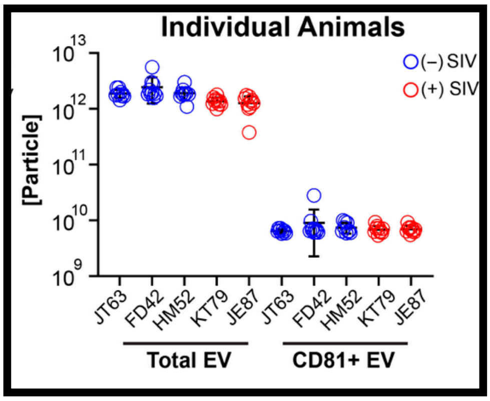
Individual Animals
(–) SIV
(+) SIV
[Particle]
JT63
FD42
HM52
KT79
JE87
Total EV
CD81+ EV

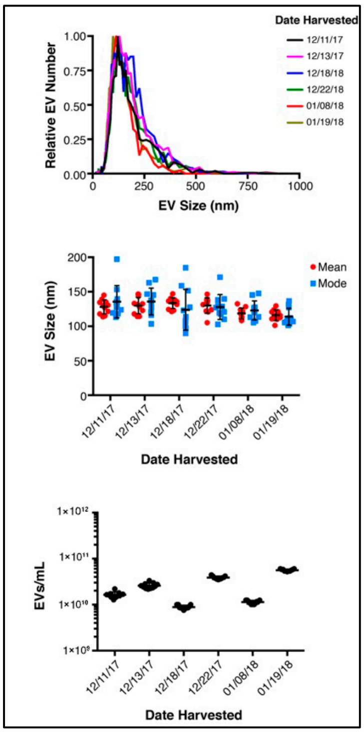
Date Harvested
12/11/17
12/13/17
12/18/18
12/22/18
01/08/18
01/19/18
Relative EV Number
EV Size (nm)
Mean
Mode
EV Size (nm)
Date Harvested
EVs/mL
Date Harvested

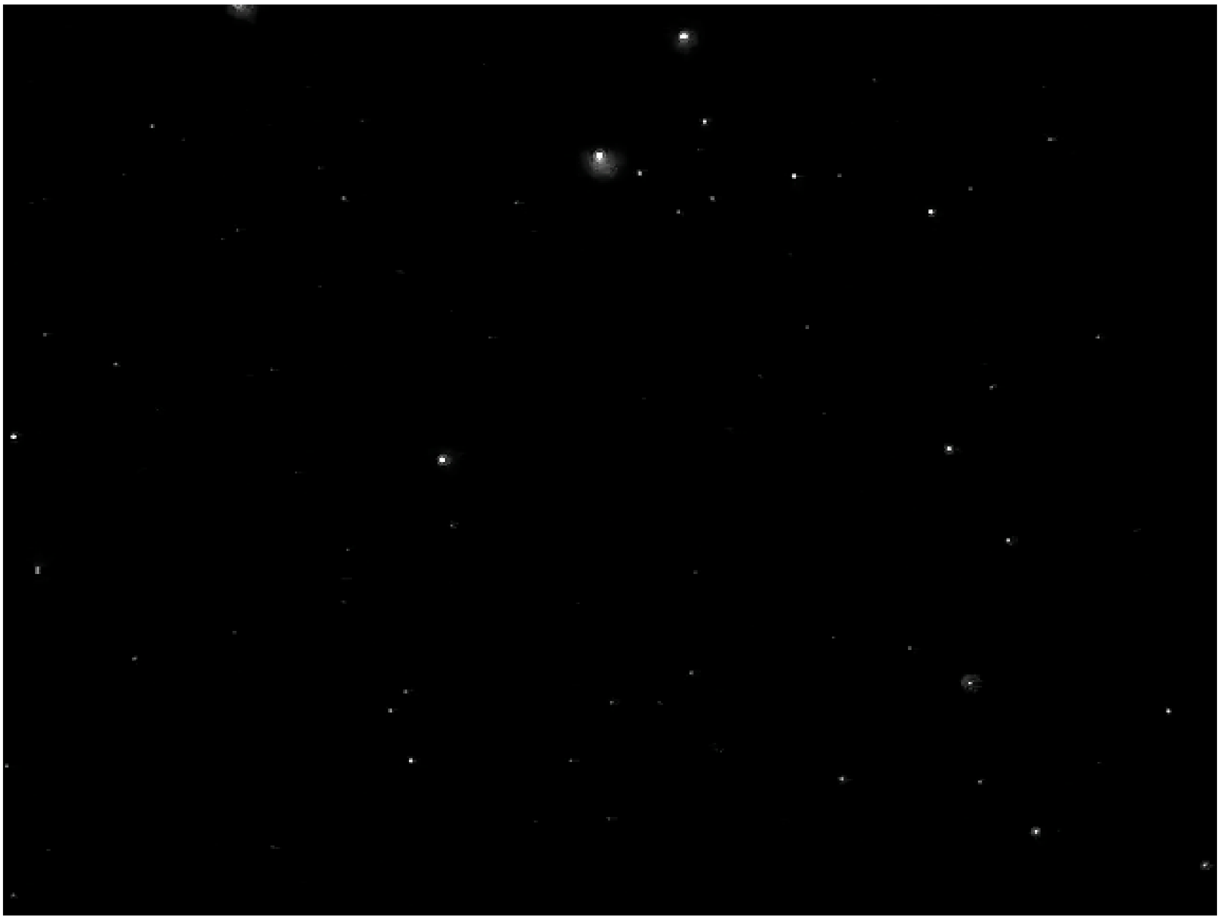

Supplemental Figure 1. Purification and characterization of extracellular vesicles (EVs). EVs were purified from plasma as is routine in the Dittmer / McNamara labs. As anticipated, CD81 +ve EVs were approximately 5% of all EVs (A). This was essentially uniform across samples collected, even if the total yield was occaisionally lower (B). Nanoparticle analysis confirmed the concentration and size distribution (C).
